# Impacts of Climate Change on Indirect Human Exposure to Pathogens and Chemicals from Agriculture

**DOI:** 10.1289/ehp.0800084

**Published:** 2008-12-10

**Authors:** Alistair B.A. Boxall, Anthony Hardy, Sabine Beulke, Tatiana Boucard, Laura Burgin, Peter D. Falloon, Philip M. Haygarth, Thomas Hutchinson, R. Sari Kovats, Giovanni Leonardi, Leonard S. Levy, Gordon Nichols, Simon A. Parsons, Laura Potts, David Stone, Edward Topp, David B. Turley, Kerry Walsh, Elizabeth M.H. Wellington, Richard J. Williams

**Affiliations:** 1 University of York, Heslington, York, United Kingdom;; 2 Central Science Laboratory, Sand Hutton, York, United Kingdom;; 3 Environment Agency, Wallingford, United Kingdom;; 4 Met Office Hadley Centre, Exeter, United Kingdom;; 5 Lancaster University, Lancaster, United Kingdom;; 6 Plymouth Marine Laboratory, Plymouth, United Kingdom;; 7 London School of Hygiene and Tropical Medicine, London, United Kingdom;; 8 Health Protection Agency, London, United Kingdom;; 9 Cranfield University, Cranfield, United Kingdom;; 10 York St. John University, York, United Kingdom;; 11 Natural England, Peterborough, United Kingdom;; 12 Agriculture and Agri-Food Canada, London, Canada;; 13 University of Warwick, Warwick, United Kingdom;; 14 Centre for Ecology and Hydrology, Wallingford, United Kingdom

**Keywords:** agriculture, climate change, environmental fate, health risks, nutrients, pathogens, pesticides

## Abstract

**Objective:**

Climate change is likely to affect the nature of pathogens and chemicals in the environment and their fate and transport. Future risks of pathogens and chemicals could therefore be very different from those of today. In this review, we assess the implications of climate change for changes in human exposures to pathogens and chemicals in agricultural systems in the United Kingdom and discuss the subsequent effects on health impacts.

**Data sources:**

In this review, we used expert input and considered literature on climate change; health effects resulting from exposure to pathogens and chemicals arising from agriculture; inputs of chemicals and pathogens to agricultural systems; and human exposure pathways for pathogens and chemicals in agricultural systems.

**Data synthesis:**

We established the current evidence base for health effects of chemicals and pathogens in the agricultural environment; determined the potential implications of climate change on chemical and pathogen inputs in agricultural systems; and explored the effects of climate change on environmental transport and fate of different contaminant types. We combined these data to assess the implications of climate change in terms of indirect human exposure to pathogens and chemicals in agricultural systems. We then developed recommendations on future research and policy changes to manage any adverse increases in risks.

**Conclusions:**

Overall, climate change is likely to increase human exposures to agricultural contaminants. The magnitude of the increases will be highly dependent on the contaminant type. Risks from many pathogens and particulate and particle-associated contaminants could increase significantly. These increases in exposure can, however, be managed for the most part through targeted research and policy changes.

Weather and climate factors are known to affect the transmission of water- and vector-borne infectious diseases as well as the transport of chemicals around the environment. Climate change may therefore have important impacts on the dispersion of pathogens and chemicals in the environment. In addition, changes in climate are likely to affect the types of pathogens occurring as well as the amounts and types of chemical used for a range of scenarios. Future risks of pathogens and chemicals could therefore be very different than today, so it is important that we begin to assess the implications of climate change for changes in human exposures to pathogens and chemicals and the subsequent health impacts in the near term and in the future. Therefore, in this review, we discuss how health risks might change by exploring the current scientific evidence for health effects resulting from environmental exposure to pathogens and chemicals arising from agriculture; the potential impacts of climate change on the inputs of chemicals and pathogens to agricultural systems; and the potential impacts of climate change on human exposure pathways to pathogens and chemicals in agricultural systems. Finally, we provide recommendations on approaches to mitigate any adverse increases in health risks.

In this review we focus on the U.K. agricultural environment, but some of the conclusions are applicable and relevant to other countries in temperate areas as well as sectors other than agriculture. We focus on environmental routes of exposure, and do not consider occupational exposure pathways or direct application of chemicals to food animals.

## Effects of Agricultural Chemicals and Pathogens on Human Health

Humans may be exposed to agriculturally derived chemicals and pathogens in the environment (i.e., air, soil, water, sediment) by a number of routes, including the consumption of crops that have been treated with pesticides or have taken up contaminants from soils; livestock that have accumulated contaminants through the food chain; fish exposed to contaminants in the aquatic environment; and groundwater and surface waters used for drinking water. Exposure may also occur via the inhalation of particulates or volatiles, or from direct contact with water bodies or agricultural soils (e.g., during recreation). The importance of each exposure pathway will depend on the pathogen or chemical type (Table 1). The main environmental pathways from the farm to the wider population will be from consumption of contaminated drinking waters and food. In the United Kingdom, vector, aerial, and direct contact pathways are currently of less importance to the general population.

Evidence for adverse human health effects from agricultural contaminants comes from epidemiologic studies (occupational and general population studies), case and outbreak reports, and toxicologic assessments. Attributing health effects in the general population to specific agricultural contaminants is often difficult because multiple chemicals and pathogens are in the environment, with multiple routes whereby they may reach humans. Some naturally occurring chemicals are also harmful (e.g., mycotoxins, microcystin), which further complicates the picture.

Although often inconclusive, several studies have associated different health outcomes with exposure to chemicals from agriculture. For example, Parkinson’s disease has been linked with exposure to pesticides (Ascherio et al. 2006), and studies have suggested that repeated exposure to low levels of organo-phosphates may result in biochemical effects in agricultural farmworkers (e.g., Lopez et al. 2007), as well as enhanced risks of certain cancers, such as leukemias or lymphoma. Studies in North America have associated chlorphenoxy herbicide exposure with circulatory and respiratory malformation, congenital abnormalities, urogenital and musculoskeletal anomalies, and changes in the male:female sex ratio of offspring (Stillerman et al. 2008).

Outbreaks of waterborne disease from the contamination of water supplies with animal waste (often associated with water treatment failures) remain an important public health problem (Crowther et al. 2002). *Cryptosporidium* transmission in humans has been linked to areas where manure is being applied to land (Lake et al. 2007a). There is also good evidence that heavy rainfall increases the risk of sporadic cases of *Cryptosporidium* in England (Lake et al. 2005, 2007b). Exposure to *Mycobacterium avium* in agricultural systems has been associated with Crohn’s disease, although the mechanism is not clear (Pickup et al. 2005).

Agricultural chemicals may also indirectly affect human health via other, often unanticipated pathways. Examples include dispersal of biotoxins into coastal communities resulting from harmful algal blooms triggered by inputs of nitrates from agriculture (Codd et al. 2005; Fleming et al. 2006; Kirkpatrick et al. 2006) and the agricultural use and environmental occurrence of antibiotics that may facilitate the selection of antibiotic resistance in microbes in the soil and water environments (Boxall et al. 2003).

## Effects of Climate Change on Chemical and Pathogen Inputs

Climate change is also likely to change the inputs of chemicals and biological contaminants to agricultural systems and may also affect their chemical form (Table 2). Changes in the abundance and seasonal activity of agricultural pests and diseases are predicted (Bloomfield et al. 2006; Cannon 1998; Food and Agriculture Organization 2008; Gale et al. 2007; Haines et al. 2000; Patterson 1995). In addition, there may be direct effects on the effectiveness of pesticides (Patterson et al. 2004). A significant increase in the use of pesticides is therefore likely, and other biocides in the future and more effective pesticides may be required in some instances (Table 2).

Climate change may also increase the production of mycotoxins in agricultural systems (Slingo et al. 2005) as well as affect the timing of exposure, distribution, quantity, and quality of aeroallergens (Beggs and Bambrick 2005; Shea et al. 2008). Warming temperatures may also promote the production of ozone, which worsens asthma (Shea et al. 2008).

Climate change may entail changes in farming practice. For example, livestock populations increasingly subjected to thermal stress and waterlogged pastures may lead to increased indoor housing of animals. This may result in an enhanced need to store and dispose of manures. Higher temperatures may facilitate the introduction of new pathogens, vectors, or hosts (Harrus and Baneth 2005), leading to increased use of biocides and veterinary medicines (Kemper 2008). Workers may be in more frequent contact with livestock in such intensive regimes, so transmission of zoonotic diseases may increase.

Changes in contaminant transport pathways may also affect contaminant inputs to agricultural systems. Flood events can transport pathogens, dioxins, heavy metals, cyanide, and hydrocarbons from a contaminated area to a noncontaminated one (e.g., Harmon and Wyatt 2008; Hilscherova et al. 2007). Climate change is likely to increase frequency of heavy precipitation events, so transport of historical contaminants from previously undisturbed sediments may occur. This could have implications for residue levels in food crops and food animals (e.g., Casteel et al. 2006). Because irrigation demands may increase because of warmer and drier summers, water of poorer quality (including partially treated wastewater) may be applied to crops, resulting in additional contaminant loadings to crops (e.g., Rose et al. 2001). Changes in temperature and precipitation could also increase aerial inputs of volatile and dust-associated contaminants. Finally, changes in bioavailability may occur, with less bioavailable forms of contaminant being converted to more bioavailable forms. For example, Booth and Zeller (2005) suggested that increases in temperature could enhance the methylation rate of mercury.

Alongside climate-change–driven changes in chemical and pathogen inputs, other drivers are also likely to affect the contaminants in agricultural systems. For example, the use of composting for treatment of municipal waste is increasing, with a portion of the resulting compost being used in agriculture. This is likely to increase loadings of microbes, heavy metals, and persistent organic pollutants in agricultural land (Deportes et al. 1995). Increases in the costs of synthetic chemicals and a move toward organic farming will result in a reduction in the amounts of agrochemicals and fertilizers applied in agriculture in the United Kingdom.

## Impacts of Climate Change on Transport, Fate, and Exposure

**Figure 1:**
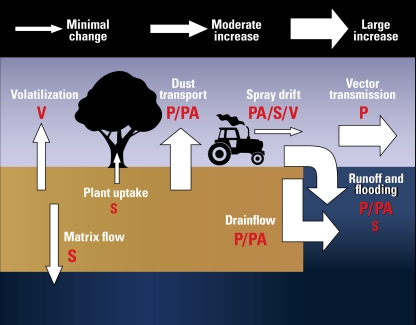
Predicted impacts of climate change on major environmental pathways for human exposure to pathogens and chemicals from agriculture. Letters indicate which contaminant classes are likely to be transported via an individual pathway: P, particulate (e.g., bacteria, viruses, spores, engineered nano-particles); PA, particle-associated (e.g., hydrophobic organics, ammonium, heavy metals); S, soluble contaminant (nitrates, group 1 and 2 elements, reactive phosphorus, hydrophilic pesticides); V, volatile contaminant. Larger and smaller letters indicate the greater and lesser extent, respectively, to which each contaminant type will be transported by the pathway (e.g., methane, nitrous oxide, ammonia, sulfides).

Both transport pathways and fate processes for chemicals and pathogens will likely be affected by changes in climate conditions, and this will affect the exposure level. The significance of a particular pathway or process depends on the underlying properties (e.g., hydrophobicity, solubility, volatility) and form of the contaminant (particulate, particle associated, dissolved; Figure 1).

### Transport to water bodies

Transport of contaminants from agricultural land to water bodies (ditches, rivers, lakes, groundwater) is well understood and depends on the soil properties and the intensity of water flow (Haygarth et al. 2000). Several studies have indicated that transport of contaminants to water bodies will increase with extreme precipitation events (e.g., Curriero et al. 2001; Kistemann et al. 2002).

Overland flow, macropore flow, and flood immersion are the most important flow routes for particulates (e.g., prions, viruses, engineered nanoparticles, bacteria, spores) and sorptive contaminants (e.g., hydrophobic organics, ammonium, heavy metals). Climate change may lead to a greater frequency of macropore and overland flow events as the infiltration capacity of the soil is exceeded. In addition, drier summers may lead to longer periods of very high soil moisture deficits, which may lead to increased hydrophobicity of soil surfaces and increased runoff during more intense summer rainfall. A secondary effect of dry summers may be an increase in soil shrinkage cracks, which may result in a more extensive and better connected macropore system.

Matrix flow is the most important route for transport of dissolved contaminants such as group 1 and 2 elements, nitrates, dissolved organic carbon, reactive phosphorus, and hydrophilic pesticides (e.g., Haygarth et al. 2000). This pathway may be influenced under climate change, but to a lesser extent than the pathways described above. Matrix flow may increase with wetter winters, resulting in higher soil moistures and hydraulic conductivities. In the summer, soil moistures may be lower and hydraulic conductivities reduced. These lower summer soil moistures may be offset by increased irrigation, thus restoring the matrix flow route temporarily, especially if the irrigation is poorly targeted.

Flood events have already been demonstrated to enhance the contamination of water bodies by pesticides (e.g., Donald et al. 2005). Flood immersion is likely to increase and aid the dispersion of agricultural chemicals after immersion in floodwater. This pathway has perhaps not received as much attention in this context as those described above.

Changes in precipitation levels and patterns will also affect river flows, changing both annual totals and seasonal patterns of flow (Falloon and Betts 2006). For rivers draining impermeable catchments, flow may decline rapidly when runoff decreases and effluent discharges are likely to make up a higher proportion of river flow. Rivers where flow is sustained by natural groundwater inflows are less sensitive to changes in runoff, although prolonged drought and depletion of ground-water may result in decreased flows that may persist even after new rainfall. Low flows may threaten effluent dilution, resulting in greater pathogen loading. However, low flows may also result in an increased amount of time between discharge and abstraction, allowing more time for pathogen and chemical decay to occur.

Models exist that account for some of these flow pathways but not necessarily for all contaminant types. Most field- or catchment-based water-quality models for agricultural systems account for vertical and horizontal matrix flow of dissolved contaminants, and some have been designed or modified to account for macro pore flow (e.g., Borah and Kalita 1999; Jarvis et al. 1997). Models also exist for surface runoff and sediment transport (e.g., Arnold et al. 1998; Ma et al. 2000).

### Transport to the air compartment

Contaminants may be transported in the atmosphere via spray drift during the application process, volatilization and dispersion from treated surfaces (e.g., plants and soils), and wind-blown dust particles from soil surfaces. Typically, a portion of a sprayed pesticide is transported away from the target area by spray drift. The extent of the spray drift depends on weather conditions, such as wind speed. Because the impacts of climate change on wind speed are uncertain, whether loss through spray drift will increase is not clear, nor is how the significance of this loss route will change relative to surface runoff or drainage. As more information becomes available on wind-speed changes, the impacts might be modeled using mechanistic models such as the program for Drift Evaluation for Field Sprayers by Computer Simulation (Holterman et al. 1997) that consider wind speed and atmospheric stability.

Volatile organic and inorganic contaminants (e.g., methane, nitrous oxide, ammonia, sulfides) can be transported from agricultural fields via a combination of volatilization and dispersion (Bloomfield et al. 2006). The extent of the transport depends on the surface temperature, air temperature, and wind speed, all of which are predicted to change as a result of climate change. Dust can be released into the atmosphere during soil tilling and crop harvesting and is an important transport pathway for particulate and particle-associated contaminants, such as bacteria, fungal and bacterial spores, steroids, pesticides, and poly-cyclic aromatic hydrocarbons (Rogge et al. 2007). Soil dust has already been linked to human health impacts (Smith and Lee 2003). The predicted hotter drier summers could lead to increased drying of soils and an increase in surface dust and hence increased transfer into the environment (Zobeck and Van Pelt 2006). Transport of dust can be predicted using empirical models (Saxton et al. 2000) through to more complex models that link meteorologic models with dust emission models (Sundram et al. 2004). Under drier conditions, bioaerosols such as fungal spores and endotoxins are likely to be more of a problem than today (Adhikari et al. 2004; Dutkiewicz et al. 2000; Pillai and Ricke 2002).

### Transport into food items

Uptake of chemicals from soil into plants depends on the physicochemical properties of the contaminant and the nature of the soil (Briggs et al. 1982; Burken and Schnoor 1998). Although warmer climates can enhance evapotranspiration and uptake, these changes will probably be offset by the effects of increased concentrations of carbon dioxide that could reduce the activity of plant stomata and reduce plant transpiration. The bioavailability of chemical contaminants to crops may also increase with the predicted decline in soil organic carbon content in the future (Bellamy et al. 2005). Selected contaminants (e.g., mercury) may also be converted from a less bioavailable form to a more bio-available form due to temperature increases (e.g., Booth and Zeller 2005). However, overall, the effect of climate change on plant uptake of chemical contaminants is likely to be small. Current regulatory models to simulate chemical fate and transport incorporate very simple routines to describe plant uptake and interception. These are probably inappropriate for estimating the impacts of climate change on human exposure from plant residues.

### Vector transport

Climate change may be accompanied by an increase in the abundance and variety of vectors and host reservoirs for human and animal pathogens. Pathways of transmission within and between populations of wildlife, livestock, and humans will potentially be enhanced (Sutherst 2004). For example, for mosquitoes, warmer temperatures will increase populations, lifespan, geographic distribution, rates of multiplication, feeding on humans, and inoculation rates and shorten the period between infection and infectivity (Chin and Welsby 2004). Water-related extreme weather events could also affect human–mosquito interactions, potentially increasing human contact and the incidence of malaria. Alongside this, increases in tick populations may occur, which might affect the incidence of Lyme disease (Ebi et al. 2006; Haines et al. 2000). However, histories of many diseases reveal that climate change is not the principal determinant of incidence and that human activities and their impact on local ecology have generally been more significant (Reiter 2001). The potential changes in exposure may in fact be offset by human strategies to avoid temperature increases (e.g., indoor living and air conditioning) (Reiter 2001).

### Effects on fate processes

As well as affecting contaminant transport, climate change is also likely to affect fate processes that determine the persistence and form of a contaminant in an environmental compartment. For chemical contaminants, biodegradation, transformation, and volatilization are expected to increase (Table 3), whereas sequestration of sorptive contaminants might decrease because soil organic carbon is predicted to decrease (Falloon et al. 2006; Smith et al. 2005). The significance of these changes on exposure will vary. For example, for contaminants moving by macropores or overland flow, biodegradation, transformation, and volatilization are unlikely to affect transported concentrations because of the high speed of the flow pathway and the low contact with the soil. However, for matrix flow, there is intimate contact with the soil and flow velocities are low, so removal of degradable and hydrophobic contaminants from the flow path could be significantly increased. Flood immersion may result in anaerobic conditions in agricultural soils, which may in turn affect the speciation, degradation, and transport of selected contaminant types. However, increased rates of degradation/transformation and accelerated breakdown of contaminants after deposition on agricultural land may reduce the concentrations of contaminants available to be transported by flooding. The impact of climate change on the behavior, viability, and fate of pathogens in the environment, and the stability and mobility of genes that encode attributes of public health significance, is much more difficult to assess because of a lack of knowledge. The various pathways and processes should not be considered in isolation because different climate-sensitive factors may have conflicting effects on human exposure.

## Predicted Impacts in Terms of Public Exposure

Based on the evidence above, both the inputs to and fate and transport of chemicals and pathogens in agricultural systems will change in response to changes in climate. Therefore, we discuss the implications of climate change on changes in exposure to three classes of contaminants (plant protection products, bacterial pathogens, and nutrients); the potential impacts of these contaminants on human health; and potential management options to ameliorate any identified increase in risk.

### Plant protection products

The use of pesticides will likely increase under climate change conditions as crop diseases become more prevalent, and as a result, loadings of pesticides in the environment will also increase. Amounts of pesticide applied to food items will therefore increase. The transport of particle-associated pesticides into water bodies will likely increase significantly, and there will be a moderate increase in the transport of hydrophilic and volatile pesticides. Complex interactions between fate processes could also affect exposures. Under scenarios with drier summers, water bodies will likely have less water, so contaminants will be less diluted. Therefore, it is likely that, with a few exceptions (e.g., nonpersistent substances where exposure may decrease because of increased degradation), concentrations in air and water will likely increase significantly. Human exposure via aerial transport (spray drift, volatilization, and transport on dust particles), drinking water, and food (from direct pesticide application onto a food item or irrigation of crops with contaminated water) is therefore likely to increase. The risks of human health effects may therefore be greater than today. Although a number of health effects have been associated with agriculture (e.g., Parkinson’s disease, other neurologic effects, and leukemia), the link to pesticide exposure is often unclear and heavily debated, so it is not possible to determine the impacts, if any, of these exposure changes on health. However, the exposure changes are manageable if policy makers anticipate and plan for them. On the regulatory side, policy makers should refine regulatory risk assessment tools to consider “new” transport routes and climate/soil/use scenarios and to apply these to new and existing pesticides to determine changes in risk. In the event that changes in risks are considered unacceptable, a number of mitigation options exist to reduce exposure that could be adopted. Possible on-farm solutions include increased tillage or incorporation (plowing) to reduce transport via macropores or overland flow, and improved management of field hydrology. More rigorous technological solutions could also be applied for cleaning up contaminated water, although this will result in increased costs to the consumer. Decision makers must ensure that policies, strategies, and measures are robust to climate scenarios as well as the socioeconomic scenarios such as the predicted increase in the U.K. population level to 71 million by 2031 (Office of National Statistics 2007). The conclusions from this case study can also be applied to other agricultural contaminants such as substances applied in animal manures (e.g., veterinary medicines) or sewage sludge (e.g., human pharmaceutical and heavy metals).

### Nutrients

Climate change and other drivers are likely to reduce the use of nutrient inputs in agriculture, but it is possible that intensification of agriculture in some areas will result in localized increases. Accompanying the climate effect, the overall projection is that fertilizer use will additionally decrease due to increased production costs and pressures to meet environmental targets such as those of the Water Framework Directive (European Communities 2000). The transport of particle and particle-associated phosphorus and ammonium to surface waters is likely to increase because of increased drain flow, overland flow, and flood immersion. Transport of dissolved phosphorus and nitrate is predicted to increase moderately. Despite the projected overall reductions in sources, the long-term exposure of water bodies is likely to worsen with the enhancement of pathways that will be able to take advantage of a generally large nutrient reservoir that exists in agricultural soils. These increases in exposure are likely to promote blooms of cyanobacteria, which may lead to the development of toxins (e.g., the liver toxin microcystin). This process will be further exacerbated by warmer temperatures in the spring and summer. Diseases associated with nitrate exposure may also become more of an issue. Potential on-farm mitigation options include technologies to improve fertilizer use and efficiency and increase tillage and incorporation. At a policy level, catchment-sensitive farming technologies should be encouraged.

### Microbial pathogens

Agricultural intensification is likely to result in increased disease pressures and greater use of antibiotics and disinfectants, which in turn could result in the selection of more antibiotic-resistant pathogens. For example, hot, dry summers may demand indoor housing of livestock, with implications for disease spread and increased need for veterinary medicines and disinfectants, which could increase selection for antibiotic-resistant bacteria. The abundance of vectors and secondary hosts (reservoirs) is likely to increase, and changes in climate conditions may well result in the appearance of “emerging” or “new” pathogens. Irrigation of food crops is likely to increase, possibly using poor-quality waters, which is likely to increase the occurrence of microbial contaminants in crop systems. Transport via preferential flow and runoff are likely to increase substantially, and flooding will increase mobility of microbial pathogens around the landscape. Therefore, environmental levels of microbial pathogens are likely to increase significantly, which may result in increased incidence of existing diseases and occurrence of new diseases. For waterborne pathogens, existing drinking water treatment and monitoring approaches will likely prevent human exposure, but exposure to pathogens in food items may well increase. It may be possible to mitigate these changes through increased tillage or incorporation, treatment of manure before application (or a move toward using manure as an energy source), and improved biosecurity practices.

## Conclusions and Recommendations

Overall, climate change is likely to increase human exposure to agricultural contaminants in the United Kingdom. The magnitude of the increases will be highly dependent on the contaminant type. The risks of many pathogens and particulate and particle-associate contaminants to human health could therefore increase significantly. These increases predicted in the U.K. agricultural environment can, however, be managed for the most part through targeted research and policy changes.

The sources of chemicals and pathogens of agricultural origin are likely to vary in the future because of climate and nonclimate factors. The potential for source variance arising from behavioral responses (e.g., intensification of management and altered patterns of chemical and manure use) will also be a compounding factor affecting the contaminant sources. Climate change is anticipated to fuel increased use of pesticides and biocides as farming practices intensify. Intensification may also lead to increased levels of occupational contact, increasing potential for zoonoses. Extreme weather events will mobilize contaminants from soils and fecal matter, potentially increasing their bioavailability.

Climate change will also affect the fate and transport of pathogens and chemical contaminants in agricultural systems. Increases in temperature and changes in moisture content are likely to reduce the persistence of chemicals and pathogens, whereas changes in hydrologic characteristics are likely to increase the potential for contaminants to be transported to water supplies. Models are available for predicting the effect of climate change on many selected pathways and processes, although models for certain transport routes (e.g., flood immersion and dust transport) may need developing or transferring from other sectors.

Overall, we anticipate that climate change will result in an increase in risks of pathogens and chemicals from agriculture to human health. As the current links between agricultural exposure and human health are unclear, it is not possible to estimate the magnitude of these changes or to conclude whether these increases in exposure are acceptable or unacceptable in terms of health end points. For chemicals, we believe that it is possible to manage many of these risk increases through better regulation, monitoring, and the development of a long-term research program. It is more difficult to predict the inputs and behavior of biological contaminants, so these may be more difficult to control than chemical substances. There are many major knowledge gaps and uncertainties, and we advocate that future work focuses on the following:

- The development of targeted surveillance schemes for presence and health effects of pathogens and chemicals arising from agriculture. This could include generation of quantitative microbial data in the environment (including nonculturable microorganisms), information on the presence and transport of anti biotic resistance genes, and data on occurrence of algal blooms and associate toxins.
- The development of future scenarios of land-use, social, technological, and economic change in order to assess how inputs of chemicals and pathogens may change into the future. Given the potential contribution of imported food as a source of disease burden and issues of traceability back to food processing, imported goods should also be considered.
- Generation of experimental data sets and models for exposure pathways (e.g., flood immersion, dust transport in air, post-application volatilization) that as yet have not been studied in any detail. Models for some of these exposure pathways already exist in other sectors, and these may be easily adapted to the agricultural systems. This work should include the development of an improved understanding of the uncertainties and limitations of climate scenario data for future agricultural contaminant fate.
- Refinement of regulatory models and procedures in light of new knowledge and existing risk assessments for contaminants need to be regularly updated.

**Figure 2:**
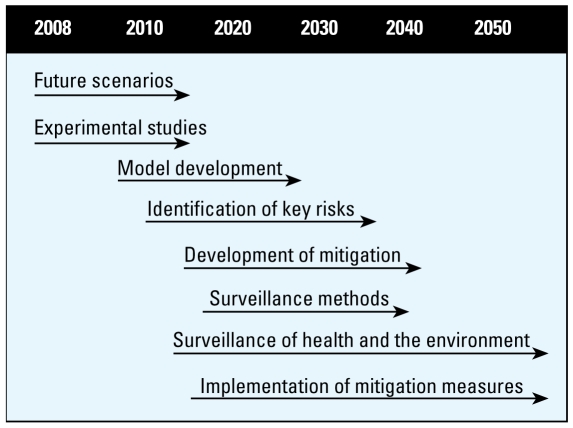
Possible time lines and strategy for research, surveillance, and risk mitigation for the predicted increases in human exposure to biological and chemical contaminants from agriculture.

This work should involve U.K. government departments and monitoring agencies (e.g., the Health Protection Agency, the Environment Agency, and Research Councils). A suggested timeline for these recommendations is provided in Figure 2.

The relationship between chemical and biological contaminants of agricultural origin and the health of the population is complex. The complexity of the relationship is increased by the projected variability of climate and extreme weather events anticipated under the climate change scenarios. Future studies into the risks of agricultural contaminants to health should therefore be multidisciplinary and pull together expertise in epidemiology, toxicology, land use, environmental chemistry, economics, and social science. Finally, it is important to recognize that agricultural systems are linked to the wider environment, and the implications of changes in inputs to and from these must not be ignored.

## Figures and Tables

**Table 1:** Potential exposure routes and health effects of chemical and biological contaminants associated with agricultural activities.

Contaminant type	Potential exposure routes	Level of knowledge of exposure	Health effects associated with exposure	Level of evidence	Degree of control in the UK	References
Chemicals						
Heavy metals (e.g., cadmium)	F	High	Renal and hepatic toxicity	I	High	a
Dioxins	F	High	Reproductive effects, carcinogenicity, immunotoxicity, endocrine disruption, neurologic effects, chloracne	I	High	d,c
Mycotoxins (e.g., aflatoxins, ochratoxins)	F	Med	Stunting of growth, liver cancers, aflatoxicosis, estrogenic effects	C	High	d
Nitrate	DW	High	Methemoglobinemia, bladder, stomach, and prostrate cancers, non_-_Hodgkin lymphoma	I	High	e,f,g
Polychlorinated biphenyls	F	High	Reproductive effects, congenital abnormalities	L	High	b,h,i
Pesticides	DW, F, A	High	Reduced eye_–_hand coordination, effects on cognitive abilities, developmental toxicity, estrogenic effects, antiandrogenic effects, congenital abnormalities, reduced stamina, birth malformations, cryptorchidism in male children, pregnancy loss, Parkinson’s disease	I	High	b,f,h,j,k
Pharmaceuticals	DW, F	Low	Estrogenic effects, carcinogenicity	L	Low	l
Phycotoxins (e.g., microcystins)	DW, RW, F	Med	Paralysis, gastrointestinal illness, amnesia, neurotoxicity, liver damage	C	High	f,m
Plant toxins (e.g., glycoalkaloids, anisatin)	F	Low	Liver cancers, cirrhosis	I	Low	m
Veterinary medicines	DW, F, A	Low	Selection of antimicrobial resistance	L	Low	n,o,p
Ozone	A	Med	Asthma	I	Low	
Bacteria and viruses						
*Cryptosporidium*	DW, RW	Med	Self-limiting diarrhea	C	High	f
*Giardia*	DW, RW	Med	Gastrointestinal illness	C	High	f
*Campylobacter*	F	Med	Gastrointestinal illness	C	Med	f
* Salmonella*	F	Med	Gastrointestinal illness	C	High	f
* Plasmodium*	V	High	Malaria	C	NA	q,r
* Borrelia*	V	Med	Lyme disease	C	High	r
Other						
Pollen	A	Med	Allergies, asthma	C	Low	s,t

Abbreviations: A, air; C, conclusive evidence linking health end point to environmental exposure; DW, drinking water; F, food; I, inconclusive evidence linking health end point to environmental exposure; L, limited evidence linking health end point to environmental exposure; Med, medium; NA, not applicable; RW, recreational water contact; V, vector borne. We have attempted, based on the available literature, to indicate the level of evidence that suggests that environmental exposure could cause the identified effect(s); we also indicate the level of control (e.g., through regulatory monitoring of food and water residues, water treatment, and requirements for risk assessment) in the United Kingdom.

a Deportes et al. (1995).

b Goldman and Koduru (2000).

c Huwe (2002).

d Hawkes and Ruel (2006).

e Cantor (1997).

f Fawell and Nieuwenhuijsen (2003).

g Boffetta and Nyberg (2003).

h Dolk and Vrijheid (2003).

i Joffe (2003).

j Donald et al. (2007).

k Stillerman et al. (2008).

l Kinney et al. (2006).

m van Egmond (2004).

n Boxall et al. (2003).

o Boxall et al. (2006).

p Hamscher et al. (2003).

q van den Berg et al. (2007).

r Githecko et al. (2000).

s Beggs and Bambrick (2005).

t Shea et al. (2008).

**Table 2:** Impacts of climate change on the inputs of chemicals and pathogens to agricultural systems.

Contaminant source	Contaminant type	Effect of climate change on input	Other drivers	Effect on input	References
Plant protection products	Herbicides, insecticides, fungicides	Increased use due to increased abundance and activity of plant diseases	Move to organic farming will reduce inputs; move to biofuels will increase inputs	High	Bloomfield et al. 2006; Cannon 1998
Fertilizers	NO_3_, PO_4_	Intensification of cropping will increase use; decreases in soil organic carbon will increase use; increased leaching may increase use; more efficient plant uptake will reduce use	Increased manufacturing costs may reduce use	Medium	—
Sewage sludge	Heavy metals, pharmaceuticals, industrial contaminants, pathogens, nutrients	Intensification of cropping will increase use; decreases in soil organic carbon will increase the need for fertilizer use	Increased economic value of biosolids may lead to lower inputs	Medium	—
Veterinary medicines	Antibacterials, parasiticides	Intensification of livestock production will increase use; increase in disease pressures will increase use	Movement of farm animals may decrease	High	Gale et al. 2007
Irrigation water	Pathogens, heavy metals, pesticides, other organic contaminants	Irrigation of crops likely to increase during dry periods	—	High	Rose et al. 2001
Flooding	Heavy metals, dioxins, polychlorinated biphenyls	Increased flooding may mobilize legacy contaminants and transport them onto agricultural land	—	Medium	Harmon and Wyatt 2008; Hilscherova et al. 2007
Vectors	Bacteria, viruses	Ranges of selected vectors change; new diseases introduced to the UK	—	High	Gale et al. 2007
Aerial deposition	Pesticides	Increased aerial transport of volatile pesticides between sites, increased soil blow	—	Medium	—
Changes in bioavailability	Dioxins, mercury, nutrients	—	—	High	Booth and Zeller 2005
Compost	Heavy metals, dioxins, polychlorinated biphenyls	—	Move to recycling increases inputs	High	Deportes et al. 1995
Contaminants from plants and bacteria	Pollen, mycotoxins	Affects distribution, quantity, and quality of autollergens; increases production of mycotoxins	—	High	Beggs and Bambrick 2005; Shea et al. 2008

We developed the assessment of effects on input based on our current knowledge.

**Table 3:** Effects of climate change on fate processes for biological and chemical contaminants.

Fate/process	Impact of climate change
Biological	
Death	Drier summers increase death rate for soil microbes Temperature extremes increase death rate Higher UV radiation levels increase death Flooding and anaerobic conditions decrease death
Growth	Increased temperature and wetness increase growth
Attenuation (loses active gene)	Uncertain
Potentiation (gene transfer)	Uncertain
Adherence	Not sensitive
Chemical	
Hydrolysis	Not sensitive
Photolysis	Increases as UV radiation increases in summer
Biodegradation/transformation	Higher temperatures increase rate Wetter winters increase rate Drier summers decrease rate
Sequestration	Lower for contaminants that sorb to soil organic matter Might be affected by drier soils Small temperature effect
Volatilization	Increases with increasing temperature
Bioconcentration	Increases with increasing temperature
Biomagnification	Not sensitive
Dilution	Increases in periods of high rainfall Decreases in prolonged dry periods

UV, ultraviolet.
